# Correlation Between Clinical Assessment and Postmortem Lung Biopsy in Patients With Pulmonary Infiltrates and Respiratory Failure

**DOI:** 10.7759/cureus.70014

**Published:** 2024-09-23

**Authors:** Chitranshu Vashishtha, Ankit Bhardwaj, Prashant M Agarwal, Chhagan Bihari

**Affiliations:** 1 Department of Gastroenterology and Hepatology, Institute of Liver and Biliary Sciences, New Delhi, IND; 2 Department of Epidemiology and Public Health, Institute of Liver and Biliary Sciences, New Delhi, IND; 3 Department of Critical Care, Institute of Liver and Biliary Sciences, New Delhi, IND; 4 Department of Pathology, Institute of Liver and Biliary Sciences, New Delhi, IND

**Keywords:** chest x-ray, liver disease, pneumonia, postmortem lung biopsy, respiratory failure

## Abstract

Background: Pulmonary complications commonly cause mortality in critically ill patients with acute and chronic liver diseases in the intensive care unit. Pneumonia is the most common clinical diagnosis supported by radiological and microbiological results, which are subjective and often with poor yield. The study aimed to correlate clinical diagnosis and postmortem lung histology in patients with liver disease with respiratory failure.

Methods: Records of acute and chronic liver disease patients with respiratory failure-associated mortality and postmortem lung biopsy from September 2009 to March 2020 were analyzed. Clinical diagnosis supported by radiological and/or microbiological data was compared with histology.

Results: One hundred eight patients (age 46.83±12.96 years), males 80 (74.1%), 63 (58.3%) cirrhosis of the liver, 30 (27.8%) acute-on-chronic-liver-failure, and 9 (8.3%) acute liver failure, were analyzed. Of the 76 patients (70.37 % of the total) with pneumonia, 33 (43.4 %) had histological evidence of pneumonia. Other histological diagnoses in these patients were normal or nonspecific changes in 27 (35.5 %) and alveolar hemorrhage in 13 (17.1 %). In the remaining 32 patients, histological diagnosis of pneumonia was evident in nine patients (28.1%). Using postmortem histology as the gold standard, the sensitivity, specificity, positive predictive value, and negative predictive value for clinical diagnosis of pneumonia were found to be 78.57%, 34.85%, 43.42%, and 71.88% respectively. The kappa statistics for agreement between the two was 0.12 (95% C.I. -0.04 to 0.27) suggesting poor agreement. Age and histological pneumonia predicted significant missed diagnosis.

Conclusion: There is poor agreement between clinical diagnosis and postmortem histology. Postmortem lung biopsy helps with the unexplained cause of death.

## Introduction

Lung diseases have a broad clinical and histological spectrum and pose unique challenges to the treating physician as the clinical and radiological signs are nonspecific. Moreover, patients with acute and chronic liver disease are at high risk of developing serious infections. Bacterial infections remain the most common cause of acute deterioration in liver function and mortality. Among various infections, pneumonia carries the maximum mortality rate (hazard ratio 2.95 (2.05-4.25)) [1]. Pneumonia is also associated with impaired mentation, renal failure, and septic shock with a high overall mortality rate [2].

However, pneumonia remains a diagnostic challenge as the clinical diagnosis, radiological findings, and microbiological methods have relatively low sensitivity and specificity. The correct distinction between the infectious and inflammatory etiology is of paramount importance as disease-specific treatment may have impact on both short- and long-term survival. The identification of the pathogen responsible for pneumonia is limited due to difficulty in obtaining tissue from the infected site. The yield may be improved by inducing the sputum, bronchoscopic lower respiratory washings, or direct lung biopsy. However, none of these procedures are usually helpful in making antemortem diagnosis [3]. The large number of organisms inhabiting the upper respiratory tract (which may not be pathogenic) further decreases the specificity. Thus, standard clinical and laboratory diagnostic tests will fail to identify the causative organism in a significant proportion of patients with pneumonia.

Postmortem examination of the lung tissue in patients with fatal pneumonia increases the overall proportion of cases with a positive diagnosis. The conventional autopsy is the gold standard method for determining the cause of death. However, it has poor acceptability by the relatives due to religious prohibitions, and concerns about the appearance of the body after the procedure and is resource intensive requiring experienced personnel and extensive laboratory support [4]. Minimally invasive tissue sampling, also known as postmortem biopsy or minimally invasive autopsy, represents a viable alternative to the conventional autopsy. This is a standardized procedure, in which tissue samples from the organs (lungs, brain, liver, or heart) and body fluids (blood, cerebrospinal fluid, ascitic fluid, or pleural fluid) are obtained with core-biopsy needles without opening the body and without any image-based guidance tools and is thus more feasible and acceptable [5].

The aim of the study was to assess the diagnostic accuracy of clinical diagnosis of pulmonary symptoms and signs using postmortem lung histology as the reference for comparison in patients with acute and chronic liver disease with respiratory failure.

## Materials and methods

This retrospective observational study was conducted at the Institute of Liver & Biliary Sciences, New Delhi, a tertiary care center, which is a national referral center dedicated to patients with liver and biliopancreatic diseases. All patients aged>18 years admitted to the intensive care unit (ICU) of the hospital with respiratory failure and evidence of diffuse infiltrates (radiographic evidence of lung infiltrates involving both the lungs) and expired subsequently and in whom a standard postmortem lung biopsy was agreed by the relatives and performed were evaluated. Patients with focal lung involvement on imaging such as chest X-ray (CXR) or computerized tomography (CT) scans or without respiratory failure were excluded. Patients with inadequate or nonrepresentative biopsy samples were also excluded. The study was approved by the institute’s ethical committee (letter number ILBS-ACAD012/8/2023-ACAD/E-12745/849) and the need for informed consent was waived due to the retrospective nature of the study.

Data collection

Data of the patients with liver disease admitted to the ICU of the hospital from September 2009 to March 2020 were retrieved from the electronic medical records and analyzed. Data on their demography, ICU diagnosis of the baseline liver disease, its severity, and the specific diagnosis of their respiratory involvement were collected. The clinical diagnosis of the underlying liver disease and the respiratory symptoms were captured from well-documented medical records.

Clinical diagnosis of liver disease

Cirrhosis of the liver was diagnosed by a combination of history, physical examination, laboratory investigations, and imaging findings of altered liver morphology and portal hypertension. Acute liver failure (ALF) was diagnosed by severe liver injury manifesting as coagulopathy with the international normalized ratio (INR) ≥ 1.5 and altered sensorium (hepatic encephalopathy) without any pre-existing liver disease, with a duration of illness of up to four weeks. Acute-on-chronic liver failure (ACLF) was diagnosed by the Asia Pacific Association for the Study of the Liver (APASL) criteria. This defines ACLF as acute hepatic insult manifesting as jaundice (serum total bilirubin ≥ 5 mg/dl) and coagulopathy (INR ≥ 1.5 or prothrombin activity < 40%) complicated within four weeks by clinical ascites and/or encephalopathy in a patient with previously diagnosed or undiagnosed chronic liver disease [6].

Clinical diagnosis of lung pathology

Respiratory failure was defined as the presence of hypoxia (SpO2 < 90%) with/without hypercapnia. Clinical diagnosis of pneumonia was made by suggestive symptoms (fever, cough, purulent sputum, breathlessness) and signs (crepitation and/or bronchial breathing on auscultation), positive culture of sputum or pulmonary specimen, along with the presence of infiltrates or consolidation in the imaging (most commonly by CXR). Pulmonary edema was diagnosed by breathlessness and cough with pink frothy sputum. On examination, tachypnoea, tachycardia, neck vein distention, and fine crepitation on auscultation favored pulmonary edema.

If the lung biopsy revealed a diagnosis that was different from the clinical diagnosis, it was considered missed diagnosis. The missed diagnoses were further subclassified into class I and class II. Class I missed diagnoses were those which if known before death would have potentially led to a change in the treatment and had led to improved survival. Class II missed diagnoses were those which if known before death would not have led to any major change in treatment or survival.

Specimen collection

The biopsy specimens were obtained from two locations in both the lungs using a 16-gauge Bard core biopsy needle. First, the skin was sterilized using alcohol swabs and then the skin was left to dry. The first site was an apical region of both the lungs by placing a needle in the supraclavicular fossa in mid clavicular site and directing the needle inferiorly. The second site was the middle lobe of each lung at the fourth intercostal space in the midclavicular line. From these two sites, three biopsy specimens were collected from both the lungs [5]. The tissue for histological examination was immediately placed in 10% neutral formalin, fixed for 4 to 24 hours, and then stored in 70% ethanol.

Histological definitions

Histological criteria of pneumonia were intense neutrophilic infiltration, fibrinous exudates, cellular necrosis, or the presence of bacteria in the pulmonary interstitium or intra-alveolar spaces. Alveoli must be at least partially filled with neutrophils, fibrinous exudates, and cellular debris. Diffuse alveolar damage was diagnosed histologically by interstitial swelling, proteinaceous alveolar edema, hemorrhage, and basement membrane disruption and denudation. Alveolar hemorrhage was diagnosed by the presence of acute hemorrhage within the alveoli and the presence of macrophages staining positive for hemosiderin [7]. Pulmonary edema was diagnosed by the presence of fluid-filled alveolar spaces suggestive of increased alveolar-capillary permeability along with variable alveolar-septal thickening [8].

Outcomes

The main objective of this study was to evaluate the accuracy of clinical diagnoses of pulmonary conditions, especially pneumonia, by comparing them with postmortem lung histology as the reference standard in patients with acute or chronic liver disease who died from respiratory failure. The frequency and predictors of major (class I) missed diagnoses based on histological findings were additional secondary objectives.

Statistical analysis

Baseline data is expressed as proportion, mean ± standard deviation, and median with an interquartile range as appropriate. Categorical variables are expressed as number (percentage). Categorical variables were analyzed by chi-squared test or Fisher exact test while the continuous variables were analyzed using an unpaired t-test or Mann-Whitney test as appropriate. The Cohen’s kappa statistic, which is a measure of agreement between two sets of observations, was calculated. Kappa values greater than 0.75 indicate excellent agreement, values between 0.4 and 0.75 indicate moderate agreement, and values less than 0.4 indicate poor agreement. To assess the factors associated independently for the class I missed diagnoses, univariate analysis was performed to find variables associated, which was followed by multivariate regression analysis. The p-value < 0.05 was considered statistically significant. All statistical tests were performed using IBM SPSS Statistics for Windows, Version 24 (Released 2016; IBM Corp., Armonk, New York, United States).

## Results

During the period from September 2009 to March 2020, a total of 153 patients with liver disease with respiratory failure and diffuse lung infiltrates underwent postmortem lung biopsy. Out of these, 45 patients were excluded (inadequate tissue 31 and nonrepresentative 14) and hence 108 patients were analyzed. Of these patients, 80 (74.07%) were males, with a mean age of 46.83 years. Cirrhosis of the liver was the most common underlying liver disease, present in 63 (58.33 %) patients, followed by ACLF which was present in 30 (27.78 %) patients. Other causes were acute liver failure (nine patients), infiltrative liver disease (three patients), post-liver transplantation (two patients), and non-cirrhotic portal fibrosis (one patient). The baseline characteristics of the patients are shown in Table 1. Pneumonia was the most common clinical diagnosis (76 patients, 70.37%). Rest was due to acute renal failure with fluid overload (five patients), pulmonary edema (seven patients), cholangitis (three patients), and increased vascular permeability due to sepsis (10 patients), and liver failure (seven patients).

**Table 1:** Baseline characteristics of the patients Categorical variables reported as absolute (n) and relative frequencies (%), while continuous variables reported as mean ± SD or median (IQR), as appropriate. BMI, body mass index; ACLF, acute-on-chronic liver failure; ALF, acute liver failure; ALT, alanine aminotransferase; AST, aspartate aminotransferase; PT (INR), prothrombin time (international normalized ratio); CTP, Chile-Turcotte-Pugh score; MELD, model for end-stage liver disease

Parameter	Overall cohort (n=108)
Age (yrs)	46.83±12.96
Gender (M: F)	80:28
BMI (kg/m^2^)	26.73±5.10
Liver disease	Cirrhosis (63)
	ACLF (30)
	ALF (9)
	Post-liver transplantation (2)
	Infiltrative liver disease (3)
	Non-cirrhotic portal fibrosis (1)
Hemoglobin (gm/dl)	10.2±2.28
Total leukocyte count (/µl)	10.6(6.75-18.35)
Platelet count (/µl)	96.5 (66.5-149.5)
Serum bilirubin (mg/dl)	12.99±10.33
ALT (U/l)	49.5 (29-89)
AST (U/l)	107 (61-183)
Serum albumin (g/dl)	2.27±0.61
Serum alkaline phosphatase (U/l)	124 (81-161)
PT (INR)	2.08 (1.59-3.13)
Serum urea (mg/dl)	40.5 (25.65-77.75)
Serum creatinine (mg/dl)	1.16 (0.74-2.08)
Serum sodium (mEq/l)	134.09±10.41
CTP score	11.87±1.38
MELD score	32.20±7.48

Of the 76 patients with pneumonia as antemortem diagnosis, 33 (43.4 %) had histological evidence of pneumonia. Other histological diagnosis in these patients were normal or non-specific changes in 27 patients (35.5 %) and alveolar hemorrhage in 13 patients (17.1 %), pulmonary edema in two patients, and acute respiratory distress syndrome in one patient with a PaO2/FiO2 ratio of 150. Of the 32 patients with clinical diagnosis other than pneumonia (29.6% of total), histological diagnosis of pneumonia was evident in nine patients (28.1%). Of the 15 patients with postmortem diagnosis of alveolar hemorrhage, 13 (86.7%) were diagnosed clinically as pneumonia. Similarly, out of 48 patients with normal or non-specific histological diagnosis, 27 (56.3%) patients had clinical diagnosis of pneumonia.

Using postmortem histology as the gold standard, the sensitivity, specificity, positive predictive value, and negative predictive value of clinical diagnosis were 78.57%, 34.85%, 43.42%, and 71.88%, respectively. The kappa statistics for agreement between the two was 0.12 (95% C.I. -0.04 to 0.27), suggesting poor agreement.

There was a total of 34 missed diagnoses (31.50 % of the total); 21 patients had class II missed diagnoses (19.4 %) and 13 (12.0 %) patients had class I missed diagnoses. There were 11 patients with histological diagnosis of pneumonia and 2 patients with pulmonary edema which were misdiagnosed clinically.

On univariate analysis, age, and histological pneumonia were predictors of class I missed diagnosis and remained the independent predictors on multivariate analysis using logistic regression (Table 2).

**Table 2:** Univariate and multivariate analysis for predictors of class I missed diagnosis BMI, body mass index; OR, odds ratio. Values of p in bold indicate p < 0.05

Variable	Univariate analysis			Multivariate analysis		
	OR	95% C.I.	p-value	OR	95%C.I.	p-value
Age (years)	1.06	1.01-1.11	0.030	1.12	1.03 – 1.22	0.005
Male gender	2.07	0.43-10.00	0.364			
BMI (kg/m^2^)	0.92	0.75-1.10	0.360			
Underlying liver disease	1.16	0.35-3.82	0.803			
Histological pneumonia	11.36	2.37-54.39	0.002	25.03	4.05- 154.65	0.001

## Discussion

In our study, there is poor agreement between clinical diagnosis and postmortem histology in patients with liver diseases with diffuse lung infiltrates. Similarly, there was a 12% rate of significant misdiagnosis which is more likely in patients with advanced age and if pneumonia is the underlying cause of infiltrates in CXR. The current study emphasizes the limitations of symptoms, signs, and CXR interpretation in critically ill cirrhotic patients which are non-specific and can be misleading.

Though CXR is the most frequently performed imaging, it is prone to errors in interpretation. One reason is that it is a two-dimensional image of a three-dimensional structure with superimposition of various organs. Moreover, abnormal findings are often subtle and non-specific. There are normal variations with age. Pneumonia can occur in the blind-spot locations, such as lung bases, and superimposed on the diaphragm [9]. This emphasizes the importance of doing lateral radiography as well but may be difficult in critically sick patients with multiple tubes and catheters. In an interesting study that aimed to analyze the impact of experience on interobserver variability in the interpretation of chest radiography, it was concluded that whereas the dense and segmental opacities were uniformly recognized correctly, there was considerable interobserver variability in the interpretation of patchy opacities. The variability persisted even among experienced physicians and medical students [10].

Failure to recognize a clinically important finding on CXR such as early pneumonia, pneumothorax, mediastinal widening, or early diagnosis of lung cancer may have a significant adverse impact. Missing an early or basilar pneumonia, apart from an increased risk of death in a critically ill liver disease patient, leads to unnecessary imaging, such as CT scans or pulmonary angiography. As shown by the current study, in patients with a high risk of poor outcomes from a missed diagnosis of pneumonia, it may be practical to initiate empirical broad-spectrum antibiotics based on local culture and sensitivity patterns while continuing diagnostic evaluation. However, equally important is to consider de-escalation and stopping the antibiotics within 72 hours if an alternative diagnosis is made.

Incorrect diagnosis of pneumonia may also harm the patients by delaying recognition and treatment of the actual disease responsible for lung infiltrates such as acute congestive heart failure, lung cancer, etc. Moreover, it also leads to unnecessary antibiotic use, their adverse effects and promotes antibiotic resistance. In a prospective cohort study of 48 hospitals in Michigan, inappropriate diagnosis of pneumonia was seen in 12% of patients. Elderly patients with dementia or altered mental status were more likely to be wrongly treated for pneumonia. Nearly 88% of them received a full course of antibiotics and had an increased risk of physician-documented adverse events due to the antibiotic [11]. Overdiagnosis of pneumonia is common due to multiple reasons. Firstly, patients with liver disease are prone to infections such as pneumonia which introduces a cognitive bias (tendency to make decisions based on the information that comes first in the mind). Secondly, the symptoms and signs of pneumonia are non-specific and overlap considerably with other cardiopulmonary diseases. Fever may be absent despite infection in liver disease patients. Thirdly, considering the increased risk of mortality in patients of liver disease with pneumonia and various studies [12] and meta-analyses [13] concluding that every hour delay in administrating antibiotics increases mortality in patients with sepsis, healthcare professionals may favor overtreating pneumonia over missing a diagnosis of pneumonia.

CT scans are much more informative in decision making but are not feasible bedside. Bedside ultrasound examination of the lung is an attractive modality and is increasingly adopted worldwide. It is a rapid ultrasound examination of the heart, lungs, liver, kidneys, and volume status of the patient by the treating intensivist directly which helps in instant decision making. However, it has a few limitations as well. It is highly operator-dependent and requires training for the procedure to be performed in difficult situations such as obesity, thoracic dressings, and pleural calcifications. Peripheral subpleural lesions can be visualized and assessed in detail, but the central lesions surrounded by artifacts due to the aerated lung are difficult to analyze. Moreover, the observations are also non-specific and need to be correlated clinically. Consolidation on ultrasound can be due to pneumonia, malignancy, atelectasis, pulmonary embolism, or acute respiratory distress syndrome [14].

In patients with unexplained mortality, autopsy is one of the most reliable methods to check the clinical diagnosis. Various studies have shown discrepancies between the autopsy and antemortem diagnosis ranging between 7% to 32% [15]. Though autopsy is a valuable tool for medical education and quality of care assessment, the rates of autopsy have declined considerably worldwide. This decline is related to multiple reasons such as cultural and religious causes, cost issues, and fear of litigation. These challenges have led to the development of new tools for evaluating the cause of death. One of them is minimally invasive tissue sampling. This involves obtaining the tissues postmortem by percutaneous biopsy without doing any incision on the skin or removing any organ, thus more acceptable to the family members. This is considerably less costly and can be performed by non-physicians as well in low-resource places. Though there are no standard protocols for performing the minimally invasive tissue sampling (MITS) procedure like autopsy, it can be done by needle biopsies of a limited number of easily assessable organs and tissues under image guidance or it can be a blind procedure if the involvement of the organ is diffuse [5]. The concordance rate of blind MITS with autopsy ranges from 60% to 70% in various studies [5,16] signifying its utility in resource-limited settings, specifically when the target organ is easily accessible (lung and liver) and diffusely involved by the disease process.

The current study also raises the possibility of performing antemortem lung biopsies in select patients of liver disease with lung pathologies remaining unexplained despite chest CT scan, sputum/ bronchoalveolar lavage analysis, serology, and blood culture with/without bronchoscopy. Patients of liver disease are frequently coagulopathic; hence it should be corrected prior to the procedure, preferably by viscoelastic tests analyzing global assessment of coagulation such as thromboelastography or rotational thromboelastometry. Whereas the deeper placed lesions are amenable to CT-guided biopsy, the peripheral or pleural-based lesions are easily suitable for ultrasound-guided tissue acquisition.

There are a few limitations of the study. This is a retrospective study; hence, it may have introduced bias. However, considering the methodology of the study, prospective studies are difficult to be performed. It is a single academic center study; hence, it may not apply to centers without the facility of postmortem biopsies. Point-of-care ultrasound was not used to evaluate the pulmonary pathologies or to target the lung biopsy site. Microbiological studies of the postmortem biopsy specimens were not performed. Thus, there may be underestimation of pneumonia as a diagnosis by postmortem lung biopsy.

## Conclusions

There is poor agreement between clinical diagnosis and postmortem histology in patients with acute and chronic liver disease with respiratory failure and diffuse pulmonary infiltrates on imaging. There is a tendency to over-diagnose pneumonia leading to antibiotic overuse, and on the other side missing pneumonia in elderly patients as well. Thus, judicious use of antibiotics is of paramount importance with de-escalating and stopping the antibiotic following the culture/sensitivity report. The study also highlights the potential role of image-guided lung biopsy in select groups of patients not improving after coagulopathy correction.
